# Frailty trajectory predicts subsequent cognitive decline: A 26‐year population‐based longitudinal cohort study

**DOI:** 10.1002/mco2.296

**Published:** 2023-06-05

**Authors:** Ruidan Li, Zheran Liu, Rendong Huang, Ye Chen, Zhigong Wei, Jingjing Wang, Ling He, Yiyan Pei, Yonglin Su, Xiaolin Hu, Xingchen Peng

**Affiliations:** ^1^ Department of Biotherapy and National Clinical Research Center for Geriatrics, Cancer Center West China Hospital Sichuan University Chengdu Sichuan China; ^2^ Hangzhou Linan Guorui Health Industry Investment Co., Ltd Hangzhou Zhejiang China; ^3^ Department of Abdominal Cancer, Cancer Center, West China Hospital Sichuan University Chengdu Sichuan China; ^4^ West China Hospital Sichuan University Chengdu Sichuan China; ^5^ West China School of Nursing, West China Hospital Sichuan University Chengdu Sichuan China

**Keywords:** cognitive function, frailty, population‐based cohort study, trajectories

## Abstract

Frailty refers to a decline in the physiological functioning of one or more organ systems. It remained unclear whether variations in the trajectory of frailty over time were associated with subsequent cognitive change. The aim of the current study was to investigate the association between frailty trajectories and subsequent cognitive decline based on the Health and Retirement Study (HRS). A total of 15,454 participants were included. The frailty trajectory was assessed using the Paulson–Lichtenberg Frailty Index, while the cognitive function was evaluated using the Langa–Weir Classification. Results showed that severe frailty was significantly associated with the subsequent decline in cognitive function (*β* [95% CI] = −0.21 [−0.40, −0.03], *p* = 0.03). In the five identified frailty trajectories, participants with mild frailty (inverted U‐shaped, *β* [95% CI] = −0.22 [−0.43, −0.02], *p* = 0.04), mild frailty (U‐shaped, *β* [95% CI] = −0.22 [−0.39, −0.06], *p* = 0.01), and frailty (β [95% CI] = −0.34 [−0.62, −0.07], *p* = 0.01) were all significantly associated with the subsequent cognition decline in the elderly. The current study suggested that monitoring and addressing frailty trajectories in older adults may be a critical approach in preventing or mitigating cognitive decline, which had significant implications for healthcare.

## Graphical abstract

The study investigated the association between frailty trajectories and cognition using a multilevel model on a large cohort of older adults from Health and Retirement Study (HRS). Results revealed that mild frailty (inverted U‐shaped and U‐shaped) and frailty were significantly associated with subsequent cognitive decline, indicating that frailty trajectories may be used to identify people at high risk of age‐related cognitive deterioration.

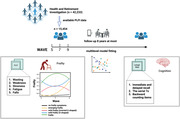

## 1 INTRODUCTION

In recent years, the global population of elderly individuals has steadily risen, leading to heightened concern regarding age‐related clinical conditions that carry significant mortality and morbidity risks.^1^, ^2^ The cognitive impairment, which has become a major burden on the healthcare system and the economy, is estimated to affect approximately 50 million people, and the number is expected to triple by 2050.^3^ The current therapeutic options for cognitive impairment are limited in their efficacy, and often carry the potential for deleterious side effects resulting from pharmacotherapy. Therefore, there is an increasing urgency for preventive measures to reduce the incidence and severity of cognitive impairment. This has become a critical issue that needs to be addressed urgently.

Frailty refers to a medical condition that is characterized by a decline in the physiological reserves of one or more organ systems, resulting in a reduced capacity to cope with physiological stressors.^4^, ^5^ The incidence of frailty appears to be highly variable across different studies and populations, and the reported prevalence rates of frailty range from low as 5% to high as 58%.^6^ Fried's frailty phenotype has been widely recognized in the identification and characterization of frailty in older adults.^7^ The five components of Fried's frailty phenotype include weight loss, limited physical activity, exhaustion, slowness, and weakness, and they reflect a broad range of physiological and functional domains that contribute to the overall concept of frailty. Based on Fried's frailty phenotype, the Paulson–Lichtenberg Frailty Index (PLFI) was found and validated to be an effective tool for assessing frailty in the Health and Retirement Study (HRS) dataset.^8^ Recent findings suggested that frailty could pose a potential risk for cognitive impairment. The results showed that elderly individuals who were frail may have a higher likelihood of developing cognitive impairments.^9^ Furthermore, there was supportive evidence indicating an association between alterations in the frailty status over time and the onset of cognitive impairment. Nari and colleagues reported that transitions between different frailty statuses (such as transitioning from non‐frail to frail) were associated with different levels of cognitive risk.^10^ Individuals who demonstrated persistent frailty had the highest risk of cognitive decline, particularly among women. In reality, the decline in frailty was a protracted and gradual process, characterized by a high degree of variability in the underlying processes of change.^11^, ^12^ Among older adults undergoing similar transitions in frailty status, there were noticeable differences in the trajectories of their frailty, indicating significant variability in the underlying processes of change. As a result, the utilization of trajectory analysis may offer a more effective means of identifying such patterns.

There may be an association between cognitive function and the frailty trajectory, according to earlier studies, and an association between the trajectory of frailty and cognitive performance. As an illustration, Howrey and colleagues implemented a group‐based trajectory model to identify and classify participants into three different frailty groups based on their unique patterns of change in frailty over time: non‐frail (*n* = 331), moderate progressive (*n* = 855), and progressive high (*n* = 149). Their subsequent analysis indicated that individuals categorized as being in the progressive high frailty group were more likely to experience a steep decline in cognitive function over time when compared to individuals in the other frailty groups.^13^ Furthermore, several other studies have reported findings that support the association between frailty and cognitive function.^14^, ^15^ Although the association between frailty and cognitive function has been widely investigated, most studies have primarily focused on examining these variables concurrently rather than exploring the potential role of frailty trajectory on later cognitive decline. As a result, there remains a significant knowledge gap regarding the association between frailty trajectory and subsequent cognitive decline. Addressing this gap will be crucial for developing effective interventions and preventive measures that can improve outcomes and quality of life in older adults who are susceptible to frailty and cognitive impairment.

In this study, we sought to enhance comprehension of the potential predictive value of frailty trajectories by utilizing a large cohort of the HRS and presenting new findings by examining diverse trajectories of frailty over time, with longitudinal changes in cognitive function as the outcome variable. To explore the association between frailty trajectories and cognitive decline in older adults, we employed a multilevel model that could account for the mutual correlation among multiple data when analyzing longitudinal data.^16^ Moreover, we conducted important subgroup analyses by stratifying the sample by gender, BMI, and loneliness, to reveal the potential heterogeneity of the relationship between frailty and cognition.

## 2 RESULTS

### 2.1 Participant characteristics

**FIGURE 1:**
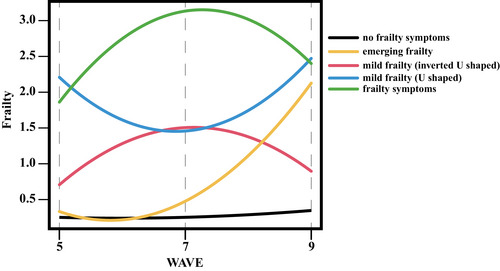
The five types of frailty trajectories of participants.

A total of 15,454 participants were included, with a maximum follow‐up period of 8 years. In our study, a total of five distinct frailty trajectories were identified and labeled as follows: no frailty symptoms, emerging frailty, mild frailty (inverted U‐shaped), mild frailty (U‐shaped), and frailty (Figure 1), which reflects the different levels of frailty severity, as well as the shape of the trajectories themselves. There were 6701, 630, 1576, 4789, and 1758 participants in these five trajectories, respectively (Table 1). Participants in the frailty trajectory tended to be older, female, with lower educational levels, and were more likely to be widowed or separated/divorced compared to those in the other frailty trajectories.

**TABLE 1:** The baseline characteristics of the included participants

Variables	Levels	No frailty symptoms (*n* = 6701)	Emerging frailty (*n* = 630)	Mild frailty (inverted U‐shaped) (*n* = 1576)	Mild frailty (U‐shaped) (*n* = 4789)	Frailty (*n* = 1758)	*p*
**Age, mean (SD)**		73.48 (6.78)	78.98 (6.59)	76.99 (6.30)	75.78 (8.18)	79.16 (7.58)	<0.01
**Gender (%)**	Male	3583 (53.5)	298 (47.3)	709 (45.0)	1645 (34.3)	558 (31.7)	<0.01
	Female	3118 (46.5)	332 (52.7)	867 (55.0)	3144 (65.7)	1200 (68.3)	
**Years of education (%)**	≤12	3896 (58.1)	380 (60.3)	975 (61.9)	3364 (70.2)	1241 (70.6)	<0.01
	>12	2753 (41.1)	246 (39.0)	587 (37.2)	1357 (28.3)	486 (27.6)	
	Missing	52 (0.8)	4 (0.6)	14 (0.9)	68 (1.4)	31 (1.8)	
**Marital status (%)**	Partnered	3321 (49.6)	351 (55.7)	696 (44.2)	1482 (30.9)	400 (22.8)	<0.01
	Without a partner	3380 (50.4)	279 (44.3)	880 (55.8)	3307 (69.1)	1358 (77.2)	
**BMI (%)**	Underweight	15 (0.2)	4 (0.6)	7 (0.4)	11 (0.2)	4 (0.2)	<0.01
	Normal weight	546 (8.1)	72 (11.4)	118 (7.5)	208 (4.3)	88 (5.0)	
	Overweight	816 (12.2)	103 (16.3)	170 (10.8)	338 (7.1)	81 (4.6)	
	Obesity	663 (9.9)	69 (11.0)	189 (12.0)	464 (9.7)	134 (7.6)	
	Missing	4661 (69.6)	382 (60.6)	1092 (69.3)	3768 (78.7)	1451 (82.5)	
**Alcohol consumption status (%)**	No	2393 (35.7)	364 (57.8)	697 (44.2)	1945 (40.6)	749 (42.6)	<0.01
	Yes	2631 (39.3)	266 (42.2)	554 (35.2)	1031 (21.5)	291 (16.6)	
	Missing	1677 (25.0)	0 (0.0)	325 (20.6)	1813 (37.9)	718 (40.8)	
**Smoking status (%)**	No	2120 (31.6)	264 (41.9)	552 (35.0)	1267 (26.5)	457 (26.0)	<0.01
	Yes	2849 (42.5)	362 (57.5)	686 (43.5)	1685 (35.2)	581 (33.0)	
	Missing	1732 (25.8)	4 (0.6)	338 (21.4)	1837 (38.4)	720 (41.0)	
**Non‐housing financial wealth (%)**	Q1	897 (13.4)	116 (18.4)	222 (14.1)	828 (17.3)	325 (18.5)	<0.01
	Q2	588 (8.8)	71 (11.3)	165 (10.5)	544 (11.4)	210 (11.9)	
	Q3	1023 (15.3)	132 (21.0)	273 (17.3)	584 (12.2)	176 (10.0)	
	Q4	1145 (17.1)	143 (22.7)	288 (18.3)	538 (11.2)	172 (9.8)	
	Q5	1370 (20.4)	168 (26.7)	303 (19.2)	483 (10.1)	157 (8.9)	
	Missing	1678 (25.0)	0 (0.0)	325 (20.6)	1812 (37.8)	718 (40.8)	
**Long‐term illness (%)**	No	4209 (62.8)	495 (78.6)	1040 (66.0)	2366 (49.4)	849 (48.3)	<0.01
	Yes	782 (11.7)	130 (20.6)	196 (12.4)	555 (11.6)	171 (9.7)	
	Missing	1710 (25.5)	5 (0.8)	340 (21.6)	1868 (39.0)	738 (42.0)	
**Loneliness, mean (SD)**		7.92 (1.41)	7.49 (1.53)	7.77 (1.49)	7.22 (1.77)	7.05 (1.66)	<0.01

Abbreviation: BMI, body mass index.

### 2.2 Association of baseline frailty with the variation of cognition

To investigate the potential association between frailty and cognitive function, univariate analysis was conducted to assess the association between baseline frailty at Wave 9 and subsequent changes in cognitive function. The findings of the univariate analysis indicated that a higher level of frailty was associated with an increased risk of cognitive impairment (*β* [95% CI] = −2.01 [−2.22, −1.80], *p* < 0.001; Table 2). After adjusting for time, basic characteristics (age, gender, years of education, marital status, non‐housing financial wealth, alcohol consumption status, smoking status, BMI), previous health condition, baseline cognitive function, and loneliness, the significant association between severe baseline frailty and a subsequent decline in cognitive function persisted (*β* [95% CI] = −0.21 [−0.40, −0.03], *p* = 0.03). This indicates that severe frailty at baseline was strongly associated with a decrease in cognitive function over time.

**TABLE 2:** Association of baseline frailty with the subsequent variation of cognition

	Changes in cognition function score [95% CI]	*p*
**Model 1**	−2.01 [−2.22, −1.80]	<0.001
**Model 2**	−2.21 [−2.42, −1.99]	<0.001
**Model 3**	−0.94 [−1.23, −0.65]	<0.001
**Model 4**	−0.95 [−1.24, −0.66]	<0.001
**Model 5**	−0.25 [−0.43, −0.07]	0.005
**Model 6**	−0.21 [−0.40, −0.03]	0.03

*Note*: Model 1 was the univariate model. Model 2 was additionally adjusted for time based on Model 1. Model 3 was additionally adjusted for basic characteristics based on Model 2. Model 4 was additionally adjusted for the previous health condition based on Model 3. Model 5 was additionally adjusted for baseline cognition function based on Model 4. Model 6 was additionally adjusted for loneliness based on Model 5.

### 2.3 Association of frailty trajectory with the variation of cognition

**FIGURE 2:**
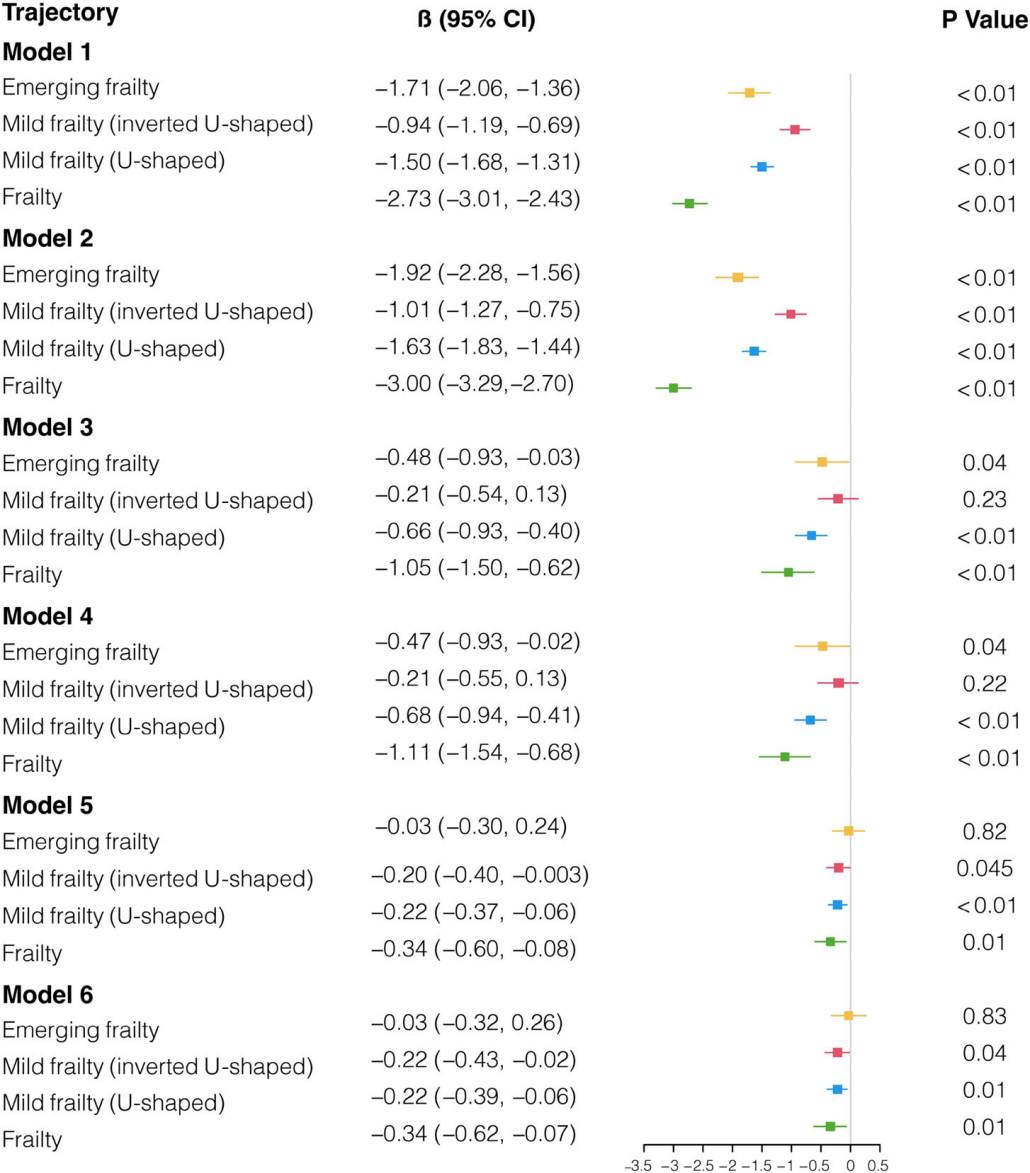
Association of frailty trajectory with the subsequent cognition decline. Model 1 was the univariate model. Model 2 was additionally adjusted for time based on Model 1. Model 3 was additionally adjusted for basic characteristics based on Model 2. Model 4 was additionally adjusted for the previous health condition based on Model 3. Model 5 was additionally adjusted for baseline cognition function based on Model 4. Model 6 was additionally adjusted for loneliness based on Model 5.

Five trajectories of frailty have been identified, namely: no frailty symptoms, emerging frailty, mild frailty (inverted U‐shaped), mild frailty (U‐shaped), and frailty. The associations between frailty trajectories and cognitive function have been determined (Figure 2). In Model 1, a univariate analysis was conducted to investigate the association between frailty trajectory and cognition. The results showed that emerging frailty (*β* [95% CI] = −1.71 [−2.06, −1.26], *p* < 0.01), mild frailty (inverted U‐shaped, *β* [95% CI] = −0.94 [−1.19, −0.69], *p* < 0.01), mild frailty (U‐shaped, *β* [95% CI] = −1.50 [−1.68, −1.31], *p* < 0.01), and frailty (*β* [95% CI] = −2.73 [−3.01, −2.43], *p* < 0.01) were significantly associated with later cognition decline. However, the association between emerging frailty and later cognitive function was no longer significant after adjusting for the time, basic characteristics, previous health condition, baseline cognition, and loneliness (*β* [95% CI] = −0.03 [−0.32, −0.26], *p* = 0.83).

Other models were developed to provide a more comprehensive understanding of how the association between frailty trajectories and cognitive function was influenced by gender, BMI, and loneliness (Table S1). In Model A, the association between frailty trajectories and cognitive function was established after adjusting for time, basic characteristics (age, years of education, marital status, non‐housing financial wealth, alcohol consumption status, smoking status), previous health condition, and baseline cognitive function. The result showed that mild frailty (U‐shaped, *β* [95% CI] = −0.18 [−0.28, −0.08], *p* < 0.01) and frailty (*β* [95% CI] = −0.25 [−0.42, −0.09], *p* < 0.01) were associated with later cognition impairment significantly. Model B was additionally adjusted for gender based on Model A. The associations between mild frailty (U‐shaped, *β* [95% CI] = −0.22 [−0.32, −0.12], *p* < 0.01) and frailty (*β* [95% CI] = −0.30 [−0.46, −0.14], *p* < 0.01) with subsequent cognitive impairment were consistent with the finding in Model A. After additionally adjusting for BMI, besides mild frailty (U‐shaped, *β* [95% CI] = −0.22 [−0.37, −0.06], *p* = 0.01) and frailty (*β* [95% CI] = −0.34 [−0.60, −0.08], *p* = 0.01), mild frailty (inverted U‐shaped, *β* [95% CI] = −0.20 [−0.39, 0.00], *p* = 0.046) was also significantly associated with later cognition impairment. The association between frailty trajectories and subsequent cognition impairment remained significant after additionally adjusting for loneliness, mild frailty (inverted U‐shaped, *β* [95% CI] = −0.22 [−0.43, −0.02], *p* = 0.03), mild frailty (U‐shaped, *β* [95% CI] = −0.22 [−0.39, −0.06], *p* < 0.01), and frailty (*β* [95% CI] = −0.34 [−0.62, −0.07], *p* = 0.01) were associated with subsequent cognition decrease.

The results revealed that participants whose frailty trajectories showed frailty, as well as mild frailty (in inverted U‐shaped and U‐shaped), exhibited an elevated risk of cognitive impairment. Furthermore, this finding emphasizes the significance of accounting for BMI as a potential confounding factor when investigating the association between frailty trajectories and cognitive function among older adults.

### 2.4 Sensitivity analysis of the association of frailty trajectory with the variation of cognition

**FIGURE 3:**
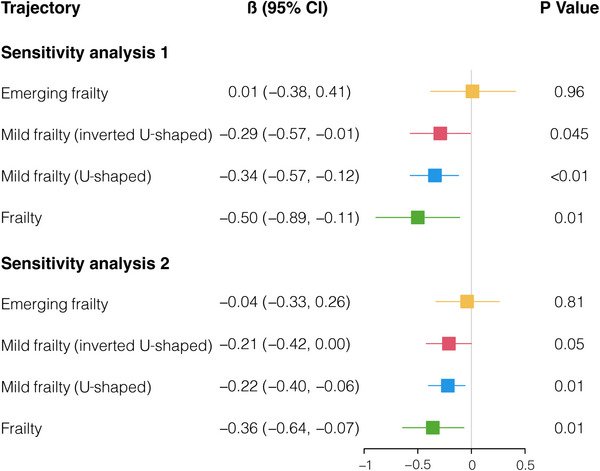
Sensitivity analyses of the association between frailty trajectory and the subsequent cognition decline. The model was adjusted for time, basic characteristics, previous health conditions, baseline cognition function, and loneliness.

To further assess the stability and reliability of the model, three sensitivity analyses were conducted (Figure 3 and Table S2). In the first sensitivity analysis, the analyses were reviewed by employing the cognitive score of participants from waves 10−13 as the outcome. The results showed that participants' frailty trajectories exhibited mild frailty (inverted U‐shaped, *β* [95% CI] = −0.29 [−0.57, −0.01], *p* = 0.045), mild frailty (U‐shaped, *β* [95% CI] = −0.34 [−0.57, −0.12], *p* < 0.01), and frailty (*β* [95% CI] = −0.50 [−0.89, −0.11], *p* = 0.01) may have a greater likelihood of suffering from cognitive impairment. Second, the participants who scored below 7 in cognitive points were removed for the second sensitivity analysis. Participants with mild frailty (inverted U‐shaped, *β* [95% CI] = −0.21 [−0.42, 0.00], *p* = 0.05), mild frailty (U‐shaped, *β* [95% CI] = −0.22 [−0.40, −0.06], *p* = 0.01), and frailty (*β* [95% CI] = −0.36 [−0.64, −0.07], *p* = 0.01) had the higher risk of cognition decline. Finally, in the third sensitivity analysis, participants who died within 2 years following the frailty evaluation were excluded, results showed that mild frailty (inverted U‐shaped, *β* [95% CI] = −0.21 [−0.42, 0.00], *p* = 0.047), mild frailty (U‐shaped, *β* [95% CI] = −0.25 [−0.42, −0.08], *p* < 0.01), and frailty (*β* [95% CI] = −0.38 [−0.67, −0.10], *p* < 0.01) were significantly associated with later cognition decline. The outcomes of these three sensitivity analyses were consistent with the primary analysis results, which indicated that the primary model in the main analysis could be applied robustly across various population cohorts.

### 2.5 Subgroup analysis of the association of frailty trajectory with the variation of cognition

**FIGURE 4:**
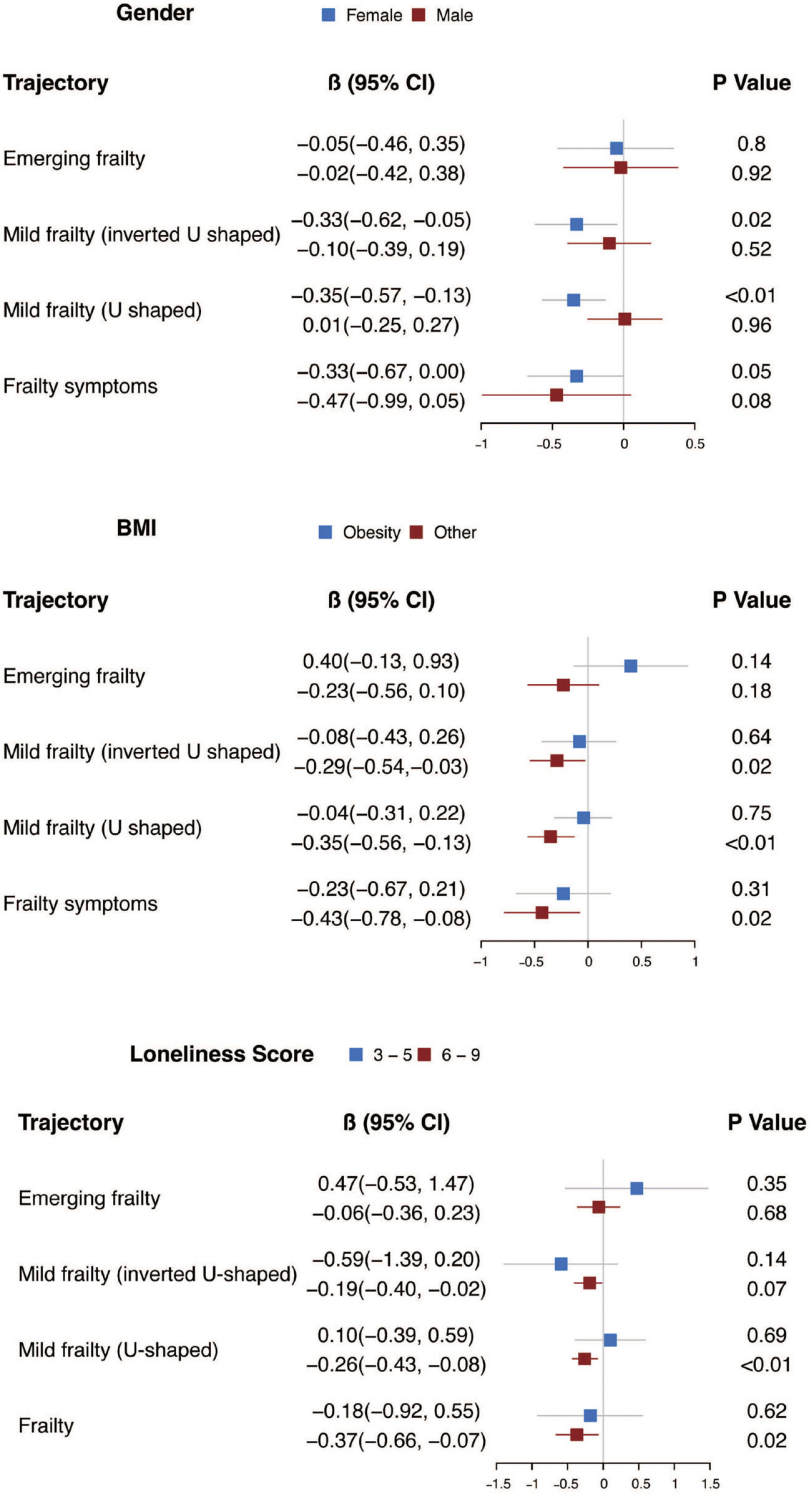
The association between frailty trajectory and subsequent cognition decline is stratified by gender, body mass index (BMI), and loneliness. The model was adjusted for time, basic characteristics, previous health conditions, baseline cognition function, and loneliness.

Gender, BMI, and loneliness were included as stratification factors in the subgroup analyses to address the potential influence of interactions between these three variables and frailty trajectory. The results of the subgroup analysis revealed that the association between frailty trajectories and declining cognitive function was more significant in females, mild frailty (inverted U‐shaped, *β* [95% CI] = −0.33 [−0.62, −0.05], *p* = 0.02), mild frailty (U‐shaped, *β* [95% CI] = −0.35 [−0.57, −0.13], *p* < 0.01), and frailty (*β* [95% CI] = −0.33 [−0.67, 0.00], *p* = 0.05) were associated with the later cognitive decline. Furthermore, this association remained consistent among participants with a non‐obese BMI, as well as those with higher loneliness scores (Figure 4).

## 3 DISCUSSION

The present study aimed to provide a thorough examination of the complex relationship between the trajectory of frailty and the following deterioration in cognitive function. The results suggested that there was a substantial association between baseline frailty and subsequent declines in cognitive function over time, with individuals exhibiting higher levels of frailty at baseline being more likely to experience a decline in cognitive function. In addition to the association between baseline frailty and cognitive decline, the present study also examined the association between specific frailty trajectories and subsequent changes in cognitive function, revealing a significant association between mild frailty (inverted U‐shaped), mild frailty (U‐shaped), and frailty and subsequent decline in cognitive function. These associations were more prominent among females, participants with a non‐obese BMI, and participants who report feelings of loneliness.

Frailty and cognitive function have been extensively studied due to their potential interconnection in the context of aging‐related decline. There appears to be a cyclical relationship between frailty and cognitive impairment, with each potentially exacerbating the other.^9^ Prior research has identified an association between cognition and frailty, as well as changes in frailty over time.^10^, ^17^, ^18^ There may be a shared pathogenic underpinning for the association.^19^, ^20^ Buchman et al. estimated the rates of change in both frailty and cognition based on the data from 2167 older adults.^14^ The majority of participants demonstrated simultaneous worsening of both frailty and cognition. Their findings highlighted a strong association between the baseline frailty level or change in frailty and cognition. This was further validated by Armstrong et al., who conducted a study on older Japanese‐American men to investigate the association between cognitive function and transition in frailty status.^17^ A total of 2817 men were recruited in the study, and the authors found a significant association between frailty status transition and cognitive decline in older adults, suggesting that cognitive decline may be a consequence of certain frailty transitions. However, it should be noted that the highly selective cohorts that were included in these two researches restricted the generalizability to the general population. Furthermore, many studies have primarily focused on the increasing frailty trajectory, rather than exploring the heterogeneity of frailty trajectories. The diversity of frailty trajectories was demonstrated in the current study. Specifically, the results showed that participants with mild frailty exhibited two different trajectory patterns, including U‐shaped and inverted U‐shaped trajectories.

Previous research suggested that females were more susceptible to cognitive impairment than males.^21^ A prior study investigated how the degree and fluctuations in frailty impact three cognitive domains including memory, speed, and executive function.^15^ This study also explored whether the association between frailty and cognition differed between males and females. The study's results showed that females may suffer from a more extensive cognitive decline than males when experiencing frailty. One of the reasons suggested by the study for that was woman has a higher level of frailty than man.^22^ In the present study, we found that there was still a gender difference in the association between frailty trajectory and cognitive function, even when individuals had the same degree of frailty. The results suggested that beyond frailty, there were other factors such as pathophysiology that may affect the gender difference in cognitive impairment.^23^

Prior studies have identified BMI as a significant factor that influenced both frailty and cognitive function in older adults. A previous meta‐analysis has demonstrated that being overweight and being underweight were both related to an elevated risk of frailty among older adults living in the community.^24^ On the other hand, the effect of BMI on cognitive function varied based on the age group, and previous studies showed that midlife underweight, obesity, and late‐life underweight increase the risk of cognitive impairment while late‐life overweight and obesity, decrease the risk of cognitive impairment.^25^, ^26^, ^27^ However, there was a dearth of evidence on how BMI affects the association between frailty and cognitive function. In this study, after adjusting for BMI, the results indicated a significant association between mild frailty (inverted U‐shaped) and later cognitive impairment. This finding underscored the need to consider BMI as a potential confounding variable when investigating the association between frailty trajectory and cognitive function in older adults. In the subgroup analyses of this study, participants who were not obese showed a strong association between frailty trajectory and cognition, whereas this effect was not prominent in obesity. This finding was consistent with previous research that suggested a higher BMI may have a protective effect in older adults, lowering the risk of cognitive impairment.

Additionally, in accordance with the prior studies, the results of the subgroup analyses involving participants with various levels of loneliness scores suggested that there was a significant and negative association between loneliness and cognitive function.^28^ Loneliness was assessed using a three‐item scale in HRS that evaluated the participant's sense of companionship, feelings of exclusion, and perceptions of social isolation (i.e., whether participants often feel they lack companionship, whether participants often feel left out, and whether participants often feel isolated from others). A previous study has demonstrated a high correlation between the total score of this three‐item loneliness scale used in HRS and the total score of the UCLA loneliness scale.^29^ So far, there is no literature that provides a definitive explanation for the cause‐and‐effect relationship between loneliness and cognitive function. According to prior research findings, there might be a reciprocal relationship between loneliness and cognitive function.^30^ Loneliness was related to cognitive decline, potentially due to reduced social interaction and limited intellectual stimulation. Conversely, cognitive impairment may also contribute to reduced social interaction, leading to increased loneliness in turn.^29^

The current study exhibits several strengths. First, the study utilized data from the HRS, a nationally representative longitudinal cohort, ensuring that the findings are generalizable to a broader population. This comprehensive investigation of the association between frailty trajectory and subsequent cognition provides important evidence for public health policy and practice. Additionally, the study examined the association between frailty and longitudinal cognition outcomes, allowing for a complete observation of evolution of the entire process and critical evidence of preventable causes of cognitive impairment.^31^ This approach is essential for understanding the complex association between frailty trajectory and cognitive decline over time, and developing effective interventions and treatments that can prevent or alleviate cognitive decline in older adults. Moreover, the present study exhibited diverse trajectories of frailty, including U‐shaped and inverted U‐shaped mild frailty, in contrast to previous studies that focused primarily on the expected worsening tendency of frailty.

The present study has some limitations. First, due to the nature of cohort studies, there might be unobserved confounding variables and residual effects that could confound the association between frailty trajectories and cognitive function. Second, the assessment of frailty in HRS was self‐reported, which may not be as precise as data obtained through clinical or laboratory tests. However, previous research has shown that the self‐reported data from HRS were comparable to objective measures in identifying frailty, which provided support for the use of self‐reported data.^7^, ^8^ Nonetheless, further research is needed to explore and validate the generalizability of these findings.

In summary, the present study has revealed the association between frailty trajectories and later cognitive function. The result showed that the associations were more pronounced among females, non‐obese individuals, and those who report feelings of loneliness, highlighting the importance of considering these demographic factors in the development of interventions and treatments for cognitive decline. Overall, the results indicated that frailty trajectories could serve as a marker for identifying people at high risk of cognitive deterioration with age. Monitoring and addressing frailty trajectories may be a crucial strategy in preventing or alleviating the cognitive decline in older adults.

## 4 MATERIALS AND METHODS

### 4.1 Participants

This study was designed based on the HRS, a nationally representative longitudinal survey of more than 37,000 individuals over age 50 in 23,000 households in the USA.^32^ The details of the HRS have been described previously.^32^ HRS was sponsored by the National Institute on Aging (NIA U01AG009740) and the Social Security Administration.

The HRS collected data by interviewing or assessing participants every 2 years. The interviews were conducted by telephone, all respondents read a confidentiality statement when first contacted, and they give verbal consent by agreeing to do the interview. This consent procedure was approved by the Michigan Institutional Review Board. The sample and data for each assessment are identified using the ordinal number of the interview wave. The HRS project was initiated in 1992 and, as of the beginning of this study, the latest available HRS data covered a population of 26 years up until 2018. The longest follow‐up time in this study spanned 8 years, from wave 9 to wave 13.

**FIGURE 5:**
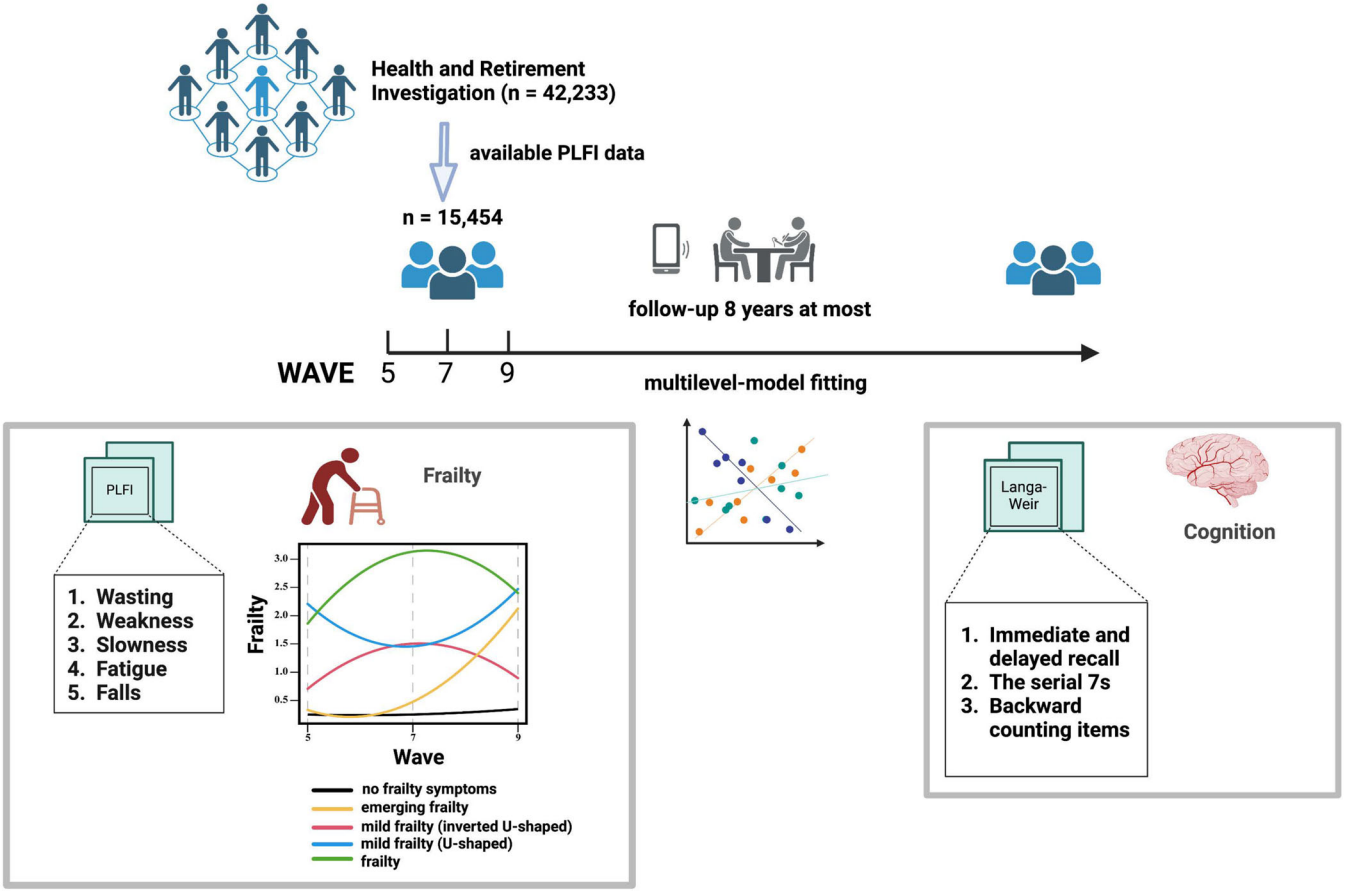
Diagrams illustrating the general study design (created with BioRender.com).

In our study, we included participants who had PLFI data available, which was the sum of the wasting, weakness, slowness, fatigue, and falls for at least one wave in waves 5, 7, or 9 (Figure 5).

### 4.2 Frailty

In the present study, we used PLFI to evaluate the frailty of participants.^8^ PLFI was validated as an effective tool for assessing frailty in the HRS. This index is the sum of the five symptoms, with a total score from 0 to 5: wasting (i.e., the individual reported the loss of at least 10% of bodyweight over 2 years), weakness (i.e., because of health problems, do participants have any difficulty with lifting or carrying weights over 10 pounds, like a heavy bag of groceries), slowness (i.e., because of a health problem, do participants have any difficulty with getting up from a chair after sitting for long periods), fatigue (i.e., have participants had any of the following persistent or troublesome problems: severe fatigue or exhaustion?), and falls (i.e., have participants fallen down in the past 2 years). If the score is in the range 3–5, the individual is deemed as frailty. The assessment for each of the five symptoms was conducted in different waves in HRS, and data for “falls” was only available during odd‐numbered waves. As a result, the PLFI data from waves 5, 7, and 9 were utilized to analyze the trajectory of frailty.

### 4.3 Cognition performance

The Langa–Weir Classification is a 27‐point scale that included immediate and delayed recall, serial 7s, and backward counting items. Respondents who scored from 0 to 6 were classified as having dementia. This classification had been validated against the prevalence of cognitive states assessed by the Aging, Demographics, and Memory Study (ADAMS) and used to evaluate the cognitive function of participants in this study.^33^ To investigate the longitudinal changes in cognitive function after PLFI assessment (waves 5, 7, 9), the Langa–Weir Classification scores from participants in waves 9–13 were determined as the study outcome, to determine the impact of frailty on cognitive function over time.

### 4.4 Covariates

Demographic and economic characteristics, lifestyle factors, and long‐term illness conditions were selected as covariates. They included age, gender (male or female), years of education (≤12 or >12), marital status (partnered or without a partner), non‐housing financial wealth (in quintile, from Q1 to Q5), alcohol consumption status (yes or no), smoking status (yes or no), body mass index (BMI, underweight, normal weight, overweight, and obesity), loneliness (mean score), and long‐term illness such as hypertension, diabetes, cancer, lung disease, heart disease, psych disease, or arthritis (yes or no).

### 4.5 Statistical analyses

Characteristics were compared by using *t*‐tests for continuous variables and chi‐square for categorical variables.

Latent class trajectory models were used to evaluate the trajectory of frailty over time. This finite mixture model is designed to identify clusters of individuals following similar progressions of some behavior or outcome over age or time.^34^ The optimal number of trajectories is usually selected based on the Bayesian information criterion (BIC). In this study, the model selection was based on the available literature, BIC, and clinical plausibility.^35^

The multilevel model was established to investigate the association between frailty trajectories and cognitive function variation. *β* (regression coefficient) and 95% confidence intervals (95% CIs) were calculated to estimate the association. Five models were constructed. Model 1 was the univariate model to analyze the association between frailty trajectory and cognition. Model 2 was additionally adjusted for time based on Model 1. Model 3 was additionally adjusted for basic characteristics including age, gender, years of education, marital status, non‐housing financial wealth, alcohol consumption status, smoking status, and BMI based on Model 2. Model 4 was additionally adjusted for the previous health condition based on Model 3. Model 5 was additionally adjusted for baseline cognitive function based on Model 4. Model 6 was additionally adjusted for loneliness based on Model 5.

To demonstrate the effects of gender, BMI, and loneliness on the association between frailty trajectories and cognitive function, we constructed four new models. Model A analyzed the association between frailty trajectory and cognition and adjusted for time, basic characteristics (age, years of education, marital status, non‐housing financial wealth, alcohol consumption status, smoking status), previous health condition, and baseline cognitive function. Model B was additionally adjusted for gender based on Model A. Model C was additionally adjusted for BMI based on Model B. Model D was additionally adjusted for loneliness based on Model C.

Three sensitivity analyses were conducted in Model 6 to test the robustness of the results from the primary analyses. In sensitivity analysis 1, we reconducted the analyses by using the cognitive function assessment data of participants from waves 10–13 as the outcome. In sensitivity analysis 2, we excluded the participants with cognition points less than 7. In sensitivity analysis 3, we excluded patients who died within 2 years after the frailty evaluation.

Subgroup analyses were conducted to show the potential influence of interactions between gender, BMI (obesity or other), and loneliness and frailty trajectory. The evaluation of loneliness was the summary score of three questions (i.e., whether participants often feel they lack companionship, whether participants often feel left out, and whether participants often feel isolated from others.), and the higher scores indicated that the participants feel more lonely.

All analyses were conducted using R 4.0.2, and a two‐sided *p*‐value less than 0.05 was considered significant statistically.

## AUTHOR CONTRIBUTIONS

Methodology, conceptualization, software, data curation, investigation, software, validation, writing—review and editing: RDL, ZRL, RDH, and YC. Conceptualization, methodology, data curation, writing—original draft, investigation, supervision, writing—review and editing: YLS, XLH, and XCP. Data curation, investigation, writing—review and editing: ZGW, LH, and YYP. All authors have read and approved the final manuscript.

## CONFLICT OF INTEREST STATEMENT

The authors declare that they have no conflicts of interest.

## ETHICS STATEMENT

Ethical approval for the HRS Study was obtained from the University of Michigan Institutional Review Board (HUM00061128), and the study has been conducted according to the principles expressed in the Declaration of Helsinki.

## Supporting information

Supporting Information

## Data Availability

The HRS database is publicly available and can be accessed by submitting a reasonable request.
