# Supplementary material for: Frailty trajectory predicts subsequent cognitive decline: A 26‐year population‐based longitudinal cohort study
Source: MedComm (2020). 2023 Jun 5;4(3):e296. doi: 10.1002/mco2.296 (PMC10242271; doi:10.1002/mco2.296)
Supplement: Supplementary file 1 — Supporting Information [file MCO2-4-e296-s001.docx]

**Frailty trajectory predicts subsequent cognitive decline: a 26-year population-based longitudinal cohort study.**

**Running title:** Frailty trajectory predicts cognition decline.

Ruidan Li ^1#^, Zheran Liu ^1#^, Rendong Huang ^2#^, Ye Chen ^3#^, Zhigong Wei ^1^, Jingjing Wang ^1^, Ling He ^1^, Yiyan Pei ^1^, Yonglin Su ^4^, Xiaolin Hu ^5*^, Xingchen Peng ^1*^

1. Department of Biotherapy and National Clinical Research Center for Geriatrics, Cancer Center, West China Hospital, Sichuan University, Chengdu 610041, Sichuan, China.

2. Hangzhou Linan Guorui Health Industry Investment Co., Ltd, Hangzhou 310000, Zhejiang, China.

3. Department of Abdominal Cancer, Cancer Center, West China Hospital, Sichuan University, Chengdu 610041, Sichuan, China.

4. West China Hospital, Sichuan University, Chengdu 610041, Sichuan, China.

5. West China School of Nursing, West China Hospital, Sichuan University, Chengdu 610041, Sichuan, China.

# Ruidan Li, Zheran Liu, Rendong Huang and Ye Chen contributed equally to this work.

* Corresponding Authors:

Prof. Xingchen Peng is to be contacted at the Department of Biotherapy, Cancer Center, West China Hospital, Sichuan University, Chengdu 610041, Sichuan, China. E-mail address: [pxx2014@163.com](mailto:pxx2014@163.com).

Prof. Xiaolin Hu is to be contacted at West China School of Nursing, West China Hospital, Sichuan University, Chengdu 610041, Sichuan, China. E-mail address: [huxiaolin@wchscu.cn](mailto:huxiaolin@wchscu.cn).

**Table. S1 Association of frailty trajectory with the subsequent variation of cognition.**

|  | **Changes in cognition function score (95%CI)** | | | | | | | | | |
| --- | --- | --- | --- | --- | --- | --- | --- | --- | --- | --- |
|  | **No frailty symptoms** | | **emerging frailty** | | **mild frailty (inverted U-shaped)** | | **mild frailty (U-shaped)** | | **frailty** | |
|  | **Changes in cognition function score (95%CI)** | ***p*** | **Changes in cognition function score (95%CI)** | ***p*** | **Changes in cognition function score (95%CI)** | ***p*** | **Changes in cognition function score (95%CI)** | ***p*** | **Changes in cognition function score (95%CI)** | ***p*** |
| **Model A** | reference | | -0.04 (-0.23, 0.14) | 0.63 | -0.07 (-0.20, 0.06) | 0.3 | -0.18 (-0.28, -0.08) | < 0.01 | -0.25 (-0.41, -0.09) | < 0.01 |
| **Model B** |  |  | -0.06 (-0.24, 0.12) | 0.53 | -0.08（-0.21, 0.05） | 0.20 | -0.22 (-0.32, -0.12) | < 0.01 | -0.30 (-0.46, -0.14) | < 0.01 |
| **Model C** |  |  | -0.03 (-0.30, 0.24) | 0.82 | -0.20 (-0.39, 0.00) | 0.046 | -0.22 (-0.37, -0.06) | 0.01 | -0.34 (-0.60, -0.08) | 0.01 |
| **Model D** |  |  | -0.03 (-0.32, 0.26) | 0.82 | -0.22 (-0.43, -0.02) | 0.03 | -0.22 (-0.39, -0.06) | < 0.01 | -0.34 (-0.62, -0.07) | 0.01 |

Model A was adjusted for time, age, years of education, marital status, non-housing financial wealth, alcohol consumption status, smoking status, previous health condition, and baseline cognition function. Model B was additionally adjusted for gender based on Model A. Model C was additionally adjusted for BMI based on Model B. Model D was additionally adjusted for loneliness based on Model C.

**Table.S2 Sensitivity analyses frailty trajectory and the subsequent variation of cognition.**

|  | **Changes in cognition function score (95%CI)** | | | | | | | | | |
| --- | --- | --- | --- | --- | --- | --- | --- | --- | --- | --- |
|  | **No frailty symptoms** | | **emerging frailty** | | **mild frailty (inverted-U shaped)** | | **mild frailty (U-shaped)** | | **frailty symptoms** | |
|  | **Changes in cognition function score (95%CI)** | ***p*** | **Changes in cognition function score (95%CI)** | ***p*** | **Changes in cognition function score (95%CI)** | ***p*** | **Changes in cognition function score (95%CI)** | ***p*** | **Changes in cognition function score (95%CI)** | ***p*** |
| **Sensitivity analysis 3** | reference | | -0.01 (-0.31, 0.28) | 0.93 | -0.21(-0.42, 0.00) | 0.047 | -0.25 (-0.42, -0.08) | < 0.01 | -0.38 (-0.67, -0.10) | < 0.01 |

Sensitivity analysis 3: Patients who died within two years after the frailty evaluation were excluded
